# The Interaction of Microbiome and Pancreas in Acute Pancreatitis

**DOI:** 10.3390/biom14010059

**Published:** 2023-12-31

**Authors:** Can Zhang, Guanqun Li, Tianqi Lu, Liwei Liu, Yuhang Sui, Rui Bai, Le Li, Bei Sun

**Affiliations:** 1 Department of Pancreatic and Biliary Surgery, The First Affiliated Hospital of Harbin Medical University, Harbin 150001, China; zhangcan@hrbmu.edu.cn (C.Z.);; 2 Key Laboratory of Hepatosplenic Surgery, Ministry of Education, Harbin 150001, China

**Keywords:** acute pancreatitis, gut microbiome, gut–pancreatic interactions, microbiome-based therapy

## Abstract

Acute pancreatitis (AP) is a common acute abdomen disease characterized by the pathological activation of digestive enzymes and the self-digestion of pancreatic acinar cells. Secondary infection and sepsis are independent prognosticators for AP progression and increased mortality. Accumulating anatomical and epidemiological evidence suggests that the dysbiosis of gut microbiota affects the etiology and severity of AP through intestinal barrier disruption, local or systemic inflammatory response, bacterial translocation, and the regulatory role of microbial metabolites in AP patients and animal models. Recent studies discussing the interactions between gut microbiota and the pancreas have opened new scopes for AP, and new therapeutic interventions that target the bacteria community have received substantial attention. This review concentrates on the alterations of gut microbiota and its roles in modulating gut–pancreas axis in AP. The potential therapies of targeting microbes as well as the major challenges of applying those interventions are explored. We expect to understand the roles of microbes in AP diagnosis and treatment.

## 1. Introduction

Acute pancreatitis (AP) is one of the most common gastrointestinal disorders worldwide. Although most cases are mild and self-limiting, 15–20% of them will progress to severe acute pancreatitis (SAP) with a mortality rate of up to 30% [1,2]. Infected pancreatic necrosis (IPN) is associated with a higher mortality rate, and more than 80% of SAP deaths are associated with enterogenic infections [3].

The digestive tract is one of the largest microbial habitats, and the overall balance of gut microbiota maintains the normal physiological function of the host. The crosstalk between the gut and pancreas participates in multiple pathophysiological processes, such as diabetes [4], pancreatic tumors [5], and pancreatitis [6]. The pancreas influences microbial composition by secreting digestive enzymes and antimicrobial peptides (AMPs) into the intestinal lumen, thereby regulating intestinal function and local homeostasis [7]. The disruption of the intestinal barrier will exacerbate intestinal bacterial translocation and enteric-derived infections, leading to the progression of AP [8]. Thus, targeting microbial alterations has emerged as a potential treatment strategy for AP.

Microbiome coevolves and changes significantly over the course of the disease, but whether gut microbiome dysbiosis is one of the causes of AP or merely a result of inflammation has not been clarified. The systematic evaluation of alteration in gut microbiota during AP and potential causes is lacking. In this review, we summarize the current research advances on the interactions of gut microbiota and AP development, with a focus on the dysbiosis of gut microbiota during AP progression and its possible mechanisms and potential novel therapeutic intervention strategies based on microbiota-related functions.

## 2. Dysregulation of Gut Microbiota during AP

Gut microbiota dysbiosis is crucial in the ‘second strike’ caused by gut-derived infections in AP. Many scholars have conducted animal and clinical studies on the relevance of microbial–pancreatic interactions to AP progression (Table 1). The ‘AP-related bacteria’ replaces the host-specific microbial community [9]. Current studies suggest altered gut microbiota in AP patients, especially SAP patients, and the abundance of opportunistic pathogens was increased, whereas beneficial bacteria such as *Bifidobacterium* and *Lactobacillus* were decreased [10]. In addition, AP patients showed an increased abundance of *Bacteroidetes*, *Proteobacteria*, *Escherichia-Shigella*, *Erysipelotrichaecease*, *Streptococcus*, and *Enterococcus* compared to healthy volunteers, while the abundances of *Firmicutes* and *Actinobacteria* were decreased [11,12,13,14]. In animal experiments, a remarkable decrease in the relative abundances of *Saccharibacteria* and *Tenericutes* at the phylum level and an increase in *Escherichia-Shigella* and *Phascolarctobacterium* at the genus level were found [15,16,17]. These studies suggest that gut microbiota plays essential roles in the progression of AP and reveal the relationship between gut microbiota and AP from multiple perspectives. By detecting the gut microbiota alteration of patients with different severity, the molecular mechanism of the “microbe–pancreas axis” regulating AP was explored, which is expected to assign microbial targets and related metabolites in the assessment of AP severity.

### 2.1. The Altered Microbial Community and Different Etiologies

Gallstone remains the leading cause of AP, followed by alcohol abuse [24], and the incidence of hypertriglyceridemia pancreatitis (HTGP) is increasing [25]. Bacteria from the gallbladder could migrate to pancreatic tissue through the lymphatic ducts and cause or exacerbate pancreatic inflammation [26]. Gut microbiota is involved in bile acid metabolism, and lifestyle affects human bile acid metabolism by regulating microbial genetics [27]. The gut microorganisms modulate bile acid synthesis and metabolism, inhibit the anti-inflammatory effects of the NF-κB pathway [28], and block Th17 cell differentiation by inhibiting RORγt [29]. However, the role of intestinal flora and bile acid metabolism in AP requires further exploration [30]. In addition, chronic alcoholic pancreatitis patients had an increased intestinal *Proteobacteria* and decreased *Bacteroidetes*, while at the genus level, *Klebsiella pneumoniae*, *Lactobacillus*, *Enterococcus*, and *Sphingomonas* were increased compared to acute alcoholic pancreatitis patients [18]. In case–control studies, acute alcoholic pancreatitis patients showed a higher level of *Actinobacteria* and decreased *Bacteroidetes* [19]. Since the comparative data with other etiologies is lacking, the evidence of alcohol-modulated gut microbiota exacerbating AP is insufficient.

The gut bacterial composition of patients with HTGP differs from other types of AP [20]. HTGP patients were associated with reduced gut microbiota abundance and microbial diversity compared to non-HTGP patients. Specifically, the abundances of *Escherichia-Shigella* and *Enterococcus* were increased, while *Dorea longicatena*, *Blautia wexlerae*, and *Bacteroides ovatus* were decreased [20,31]. Our recent study also observed that HTGP patients had a lower diversity of gut microbiota and a lack of beneficial bacteria. The abundances of *Enterococcaceae* and *Escherichia Shigella* were higher, while the abundances of *Bacteroides* and *Faecalibacterium* were lower. Correlation analysis showed that the abundances of *Faecalibacterium* and *Bacteroides uniformis* were negatively associated with disease severity [21]. These suggest that different type-dependent bacteria species affect AP development, and the unique gut microbial identity may determine the different outcomes.

### 2.2. Differences in the Gut Microbiota and Disease Severity

Gut microbiota alteration differed with the disease severity in both human and animal models [6,10,22]. In patients, the levels of opportunistic pathogens, including *Enterobacterium* and *Enterococcus* were significantly increased in SAP patients, while the levels of beneficial bacteria such as *Bifidobacteria* were decreased [22]. The SAP group showed increased *Enterobacter* and *Enterococcus* levels, respectively, while the levels of *Bifidobacterium* were decreased compared to the MAP group. In animal studies, higher abundances of *Enterobacteriaceae* and *Enterococcus*, and a lower abundance of *Bacteroidetes* were found in SAP mice [22]. Infectious acute necrotizing pancreatitis (ANP) is a devastating subgroup of SAP, of which microbial diversity was reduced and a higher abundance of *Enterobacteriaceae*, but lower abundances of *Clostridium* and *Bacteroidetes* were found compared to non-ANP groups [23]. *Enterococcus*, which can adhere to and invade the host cell surface, cross the host epithelial barrier, and enter internal organs [22,32], was found in both drainage fluid and peripheral blood of SAP patients by culture and molecular techniques. These suggest that the transferred *Enterococcus* from the gut to the pancreas and systemic circulation is a causative factor in the deterioration of the disease [16,32].

Of interest is the ability to predict patients with AP who will subsequently develop SAP and its associated complications through their fecal microbiota. Alterations in intestinal bacterial taxonomy were associated with the severity of illness, ICU admission rate, and overall prognosis of AP patients. *Enterococcus faecalis* and *Finegoldia magnas* can be used as predictors of IPN and disease severity [23]. Future studies in larger prospective multicenter projects will overcome the limitations of existing studies to understand the progression of AP disease from the perspective of the intestinal flora. This may provide new perspectives for early prediction of disease severity in AP patients as well as rational interventions.

## 3. Interactions between the Microbiome and Pancreas in AP Progression

**Figure 1:**
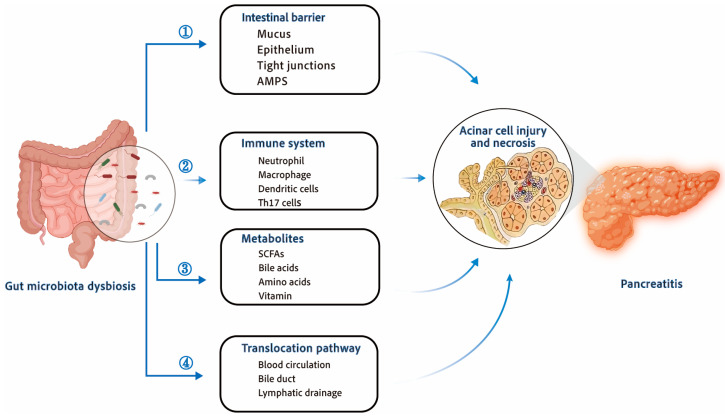
Interactions between the development of acute pancreatitis and gut microbiota dysbiosis. Dysbiosis of gut microbiota during AP destroys gut homeostasis and leads to disease exacerbation. ① Gut microbial dysbiosis will aggravate the damage of intestinal barrier, which is manifested by reduced secretion of AMPs, damaged mucus barrier, damaged epithelial cells, damaged tight connections between intestinal epithelial cells, and increased intestinal leakage. ② Gut dysbiosis affects the immune system, promoting the activation and recruitment of neutrophils and macrophages, and the activation of dendritic cells and T cells further amplifies the inflammatory response. ③ Metabolites of gut microbiota, such as SCFAs, bile acid metabolites, amino acids metabolites, etc., can affect pancreatic inflammation and regulate the progression of AP. ④ Bacteria, metabolites, and toxins can migrate to the pancreas through blood circulation, pancreatic duct, and lymphatic drainage, and affect the progression of AP through the interaction between the intestine and pancreas. AMPs—antimicrobial peptides; SCFAs—short-chain fatty acids.

The implementation of the Human Microbiome Project has contributed to the rapid development of the field of gut microbiology. Microbes mediate bidirectional communication between the intestine and organs such as the liver and lungs that affect the development of alcoholic liver disease, cirrhosis, and chronic obstructive pulmonary disease [33,34]. Targeting these gut–organ axes to treat diseases outside the gut is proving effective [35,36]. Similarly, interactions between the gut and pancreas have been studied in both pancreatic diseases and normal physiological states [37]. These findings support the hypothesis that the “gut–pancreas axis” determines the onset and progression of AP (Figure 1).

### 3.1. Intestinal Barrier Damage and Gut Microbiota Translocation

Gut barrier dysfunction is present in 60% of AP patients and is associated with worse clinical outcomes [38]. Microcirculatory damage and bacterial dysbiosis could disrupt the gut’s biological, mechanical, and immune barriers during AP [39,40]. The possible mechanism of gut barrier dysfunction may include intestinal dysmotility [41], ischemia-reperfusion injury [42], oxidative stress [43], and immune dysfunction [44]. Gut microbiota shifts through the disrupted intestinal barrier, exacerbating systemic or local inflammation and causing secondary infections. An increased abundance of *Escherichim-Shigella* in the gut improves serum levels of pro-inflammatory cytokines (such as IL-6) and further increases intestinal permeability [22]. In addition, the commensal *E. coli* MG1655 aggravates intestinal epithelial injury through TLR4/MyD88/p38 MAPK signaling [45]. Epithelial apoptosis increases during AP and damages the intestinal barrier by down-regulating tight junction proteins, such as zonula occludens-1 (ZO-1), claudin, and occludin [46,47]. Regardless of damaging the epithelial barrier, AP-induced inflammatory response damages the mucin-secreting goblet cells, which causes the injury of the intestinal mucous membrane [46]. In addition, AP exacerbates intestinal inflammation and damage by upregulating pro-inflammatory intestinal factors, including tumor necrosis factor (TNF-α) and interleukin (IL-6) [48]. Bacterial translocation following the impaired intestinal barrier is a major cause of pancreatic tissue necrotic infection and subsequent sepsis in SAP patients [49,50].

The exact mechanism of intestinal microbial dysbiosis and bacterial translocation in AP patients is unclear, but impaired intestinal homeostasis is certainly a prerequisite for bacterial translocation. Short chain fatty-acids (SCFAs) synthesized by anaerobic bacteria such as *Bifidobacterium*, *Lactobacillus*, *Bacteroides*, and *Fusobacterium* that provide energy for intestinal mucosal cells, regulate intestinal pH, maintain the integrity of tight junction proteins in intestinal mucosal epithelial cells, and improve the gut biologic and mechanical barrier function [6,51]. Gut microbiota dysbiosis caused by *Escherichia-Shigella*, *Enterococcus*, or *Staphylococcus* activates Treg cells, leading to a Treg/Th17 imbalance and disrupting the functional integrity of the gut immune barrier, which causes secondary pancreatic necrotic tissue infections [52]. Considering the important role of intestinal barrier integrity in preventing the translocation of gut pathogens to the pancreas, the specific mechanisms of intestinal barrier damage during AP deserve to be thoroughly investigated. Directions for future studies include the effects of AP on the intestinal biological, immune, and mechanical barriers and their underlying mechanisms.

### 3.2. Gut Microbiota-Derived Metabolites and AP Progression

Most physiological effects of the gut microbiota on the host organism are achieved through microbial metabolites. Gut bacteria are implicated in the metabolism of many nutrients, including SCFAs and bile acids, as well as the synthesis of several nutrients required by the body, such as vitamins and many amino acids. SCFAs are crucial agents in maintaining the functional homeostasis of the intestine [53,54]. Their primary roles include energy supply to the intestinal epithelium, intestinal barrier protection, intestinal immune regulation, and gut microbiota regulation [55]. It has been found that a decrease in butyric acid-producing strains exacerbated acute necrotizing pancreatitis and altered intestinal metabolism by regulating the synthesis and metabolism of SCFAs. Butyric acid may exert anti-inflammatory effects via the STAT1/AP1-NLRP3 pathway by inhibiting HDAC1 [12]. In addition, *Parabacteroides* produced acetate can alleviate heparanase-exacerbated AP by reducing neutrophil infiltration [56]. *Bifidobacterium* spp. and its metabolite lactate can alleviate AP through modulating the TLR4/MyD88 and NLRP3/Caspase1 pathways [57]. In addition, butyrate could alleviate pancreatic damage during AP by eliminating inflammatory factors and inhibiting NLRP3 inflammatory vesicles [54]. Microbiota-derived metabolites may be substantial messengers for gut–pancreas interactions. The advanced metabolomics and detection methods assist in bettering our understanding of the effects of gut microbiota on AP from the perspective of metabolism. Future microbial studies need to consider the alteration of gut microbiota structure, as well as the change of metabolism pattern of gut microbiota.

### 3.3. Tissue-Resident Microbes and AP

Microbiome exists in normal, non-pathological pancreatic tissue, although findings on microbial composition are inconsistent [58,59,60]. Current studies revealed the existence of bacteria within pancreatic tissue with a highly similar profile to that of the duodenum [58,61]. The predominance of *Acidaminococcus*, *Escherichia*, *Bacteroides*, and *Shigella* species, were identified from pancreatic cyst fluid [62]. In pancreatic cancer, bacteria within pancreatic tissues are tumor-specific [63] and exhibit host–microbiome interactions that affect the local immune microenvironment and patient survival [64]. The molecular mechanisms and pathways that microbial invasion into the pancreas are intriguing topics and much of the current research is focused on the field of pancreatic tumors, with limited knowledge of the pancreatic tissue-resident bacteria during AP. The possible routes of microbiota invasion into the pancreas include pancreatic ducts, blood flow, lymphatic drainage, and ascites transmural transfer. Bacteria within pancreatic tissue have been originated from the gut [65]. The fluorescence was observed in the pancreas tissue after the administration of fluorescently labeled *Enterococcus faecalis* in the mice, suggesting that the bacteria migrated from the intestine into the pancreas [58]. *E. coli* increased in the peripheral blood and was detected in the pancreas when the intestinal barrier was disrupted during AP [66]. It was also observed that *E. coli* was present in the pancreatic tissues of mice with severe pancreatic injury and infection, whereas *E. coli* was absent from the pancreatic tissues of germ-free mice [6]. These imply that the pancreatic tissue-resident bacteria may originate from the intestines via the peripheral blood and play a key factor in the progression of AP. However, the roles of pancreatic tissue-resident microbes in the development of AP, and the optional bystander or a participant in driving disease progression are unclear.

### 3.4. Microbiota and Immune Modulation

**Figure 2:**
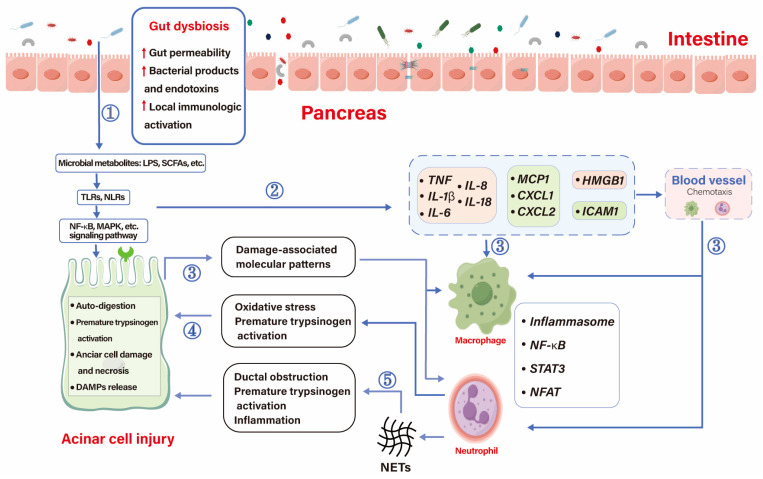
Initiation and immunoinflammatory processes influenced by AP. ① Gut dysbiosis leads to increased permeability, local immune activation, and translocation of bacteria and their products and toxins, such as SCFAs and LPS, which aggravate acinar cell damage through interaction with signaling pathways on acinar cells. ② Bacteria, metabolites, and damaged acinar cells promote the release of cytokines, chemokines, adhesion molecules, and injury-related molecular patterns that recruit immune cells to the pancreas. ③ Once recruited, chemokines, cytokines, and damage-associated molecular patterns around acinar cells activate immune cells and amplify the inflammatory response. ④ Macrophages amplify the inflammatory response through NF-κB, STAT3, neutrophils cause premature activation of trypsin, and acinar cells injury through oxidative stress. ⑤ Neutrophils can also cause duct obstruction, premature activation of pancreatic enzymes and amplify inflammatory responses by releasing NETs. 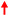, up-regulation; CXCL, chemokine ligands; HMGB-1, high mobility group box chromosomal protein 1; ICAM1, intercellular cell adhesion molecule-1; LPS, lipopolysaccharides; MCP1, monocyte chemoattractant protein-1; NETS, neutrophil extracellular traps; NFAT, nuclear factor of activated T cells; NLRs, Nod-like receptor; NF-κB, nuclear factor kapa B; SCFAs, short-chain fatty acids; STAT3, signal sensor transcription and activation 3; TLRs, Toll-like receptors.

Gut microbiota and the human immune system have often co-evolved and complemented [67]. Gut microbiota-dependent early pancreatic auto-digestion and the release of pathogen-associated molecular patterns (PAMPs) activate local innate and adaptive immunity, which amplifies the inflammatory process in AP (Figure 2). Gut bacteria train the innate immune system, which can be activated by pattern recognition receptors (PRRs) that recognize PAMPs [68]. Gut microbiota and its derivatives, like SCFAs, bile salts, LPS, and even bacterial DNA, can effectively bind to PRRs to activate TLRs that participate in the inflammatory process [69].

Lipopolysaccharides, a component of intestinal microbes, could activate the host’s innate immune system through PRRs such as TLRs. Knocking out TLR4 could alleviate the severity and protect other organs from inflammatory damage in AP mice [70,71]. Similarly, the exhaustion of gut microbiota by antibiotics before the induction of AP inhibited the TLR9 signaling pathway and alleviated AP in mouse models [72]. In addition, T cells play a crucial role in the counterbalance against systemic inflammatory response syndrome during AP. The prophylactic T cell depletion stabilizes the intestinal immunological barrier function of Th17 cells and CD8/γδTCR IELs that develop less bacterial translocation to the pancreas [52]. The gut microbiota overcomes the gut barrier and translocates to the pancreas which activates the host immune response and triggers an inflammatory cascade response during AP. Bacteria and their metabolites activate nucleotide-binding oligomerization domain 1 (NOD1) and promote the expression of nuclear factor-κB (NF-κB) and type I interferons in pancreatic acinar cells, which recruit neutrophils and macrophages to the pancreatic localization and accelerate the progression of AP [73].

On the other hand, excessive inflammatory activation and cascade amplification are important aspects of AP development and disease progression. Neutrophils and macrophages assume key roles in the amplification of AP. *Parabacteroides* could alleviate cerulein-induced AP in mice through producing acetate to reduce neutrophil infiltration [56]. Probiotics have a positive anti-inflammatory effect by reducing the synthesis and secretion of TNF-α and INF-γ in the intestinal mucosa. The beneficial intestinal bacteria could mitigate AP and inhibit the inflammatory response through NLRP3, TLR4, and p38MAPK inflammatory signaling pathways [45,74]. The increased *Clostridium lituseburense* could reduce blood levels of inflammatory cytokines such as IL-1b, TNF-α, CXCL1, and IL-18, which in turn reduced the severity of AP and the incidence of infectious complications [75]. Conversely, an increased abundance of pathogenic bacteria in the gut could exacerbate pancreatic and systemic inflammation in mice [22]. The expression levels of pro-inflammatory factors like IL-1, IL-8, and TNF-α in SAP patients were positively correlated with levels of intestinal aerobic bacteria including *Enterobacter* and *Enterococcus* [10]. The intestinal NLRP3 inflammatory vesicle activation contributes to pancreatic injury, and knockdown of the NLRP3 gene increases *Lactobacillus* and decreases *Shigella*, which enhance intestinal mucosal barrier function and the remission AP [6].

## 4. Potential Strategies for Targeting the Microbiota–Pancreatic Axis in the Treatment of AP

**Figure 3:**
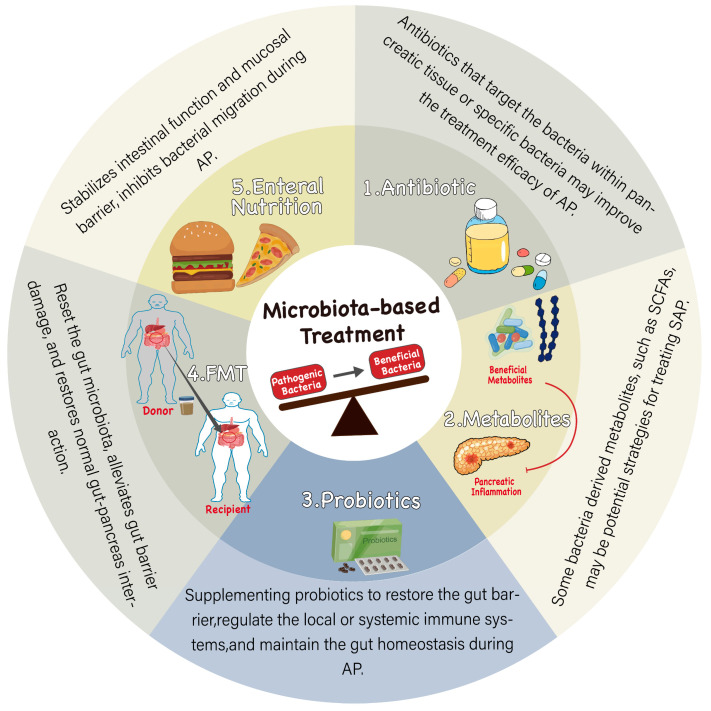
Microbiota-based treatments for AP. Restoration of intestinal microecological balance reduces gut inflammation and protects the gut barrier. 1. Antibiotics are the most direct way to regulate gut microbiota, inhibit the overall bacteria, target specific harmful bacteria, or reduce pathogenic bacteria translocating to the pancreas, which may improve the treatment of AP. 2. Supplementation of bacterial metabolites such as short-chain fatty acids may be an effective strategy to improve SAP. 3. Probiotic therapy supplements beneficial intestinal flora to partially improve intestinal imbalance, repair gut barrier, regulate systemic immunity, and thus improve the treatment of AP. 4. FMT transplants the whole gut microbiota of healthy people into the intestine of AP patients to reset their gut microbiota, improve gut imbalance, maintain normal intestinal function, and then regulate the pancreas. 5. Enteral nutrition has been shown to contribute to the early recovery of intestinal function. Special nutrients may help maintain normal intestinal function; on the other hand, it may improve the treatment of AP by affecting gut microbiota and reducing bacterial translocation.

Microbiome-based therapies mainly focus on modulating gut microbial community structure and the level of microbiota-derived metabolites, such as probiotics, antibiotics, FMT, and enteral nutrition (Figure 3). These therapeutic strategies eliminate pathogenic bacteria by supplementation with specific beneficial organisms and their derivatives or resetting the whole flora. Targeting the gut microbiota is a potential treatment option to alleviate bacterial translocation and inflammation during AP.

Microbiome-based therapies are potentially effective in animal studies, but only a small number have advanced to preliminary clinical trials. In a double-blind, randomized clinical study (RCT), Wan et al. [76] investigated the application value of probiotics in reducing the length of hospital stay in mild AP patients and found that probiotics supplementation may be safe and effective in reducing the length of hospital stay in patients with mild AP. However, there are many kinds of probiotics, and the clinical therapeutic effects and adverse reactions are different. A multicenter, large-scale RCT study found that probiotic combination treatment failed to reduce the incidence of infectious complications in SAP patients and increased the risk of death [77]. Antibiotics are considered an effective strategy to regulate the gut microbiome, and early clinical trials have shown that prophylactic antibiotic therapy significantly reduced the incidence of infectious pancreatic necrosis [78]. However, a recent RCT study showed that prophylactic antibiotic use did not significantly reduce the risk of mortality and pancreatic infection [79]. Even worse, a recent RCT study in Japan suggests that the early prophylactic use of antibiotics has no significant clinical benefit for SAP patients but may increase the risk of hospital-acquired infections [80]. Early enteral nutrition helps maintain the integrity of the intestinal barrier and prevents bacterial overgrowth. Several RCT studies have explored the effect of the timing, type, and mode of nutritional therapy on AP treatment and compared the effects of probiotic-enhanced nutritional therapy, but from the perspective of safety, the current research results are inconsistent. Although FMT and the supplementation of metabolite have achieved impressive results for some diseases, in terms of AP, they have not entered clinical trials. Currently, microbiota-based AP treatment strategies are mostly limited to animal experiments and mechanism studies, and more clinical trials are needed in the future to evaluate the efficacy and safety of targeted intestinal microbiota treatment strategies for AP.

### 4.1. Probiotic

The use of probiotics in AP is still controversial. Four modes of probiotic contribution to human health are currently considered: (1) some specific strains (e.g., Lactobacillus and Bifidobacterium) can act as microbial barriers to directly eliminate or inhibit pathogenic bacteria [81]; (2) probiotic bacteria strengthen the intestinal barrier in a variety of ways, such as induction of intestinal mucus and antimicrobial peptides production [82,83]; (3) probiotics have local and systemic modulatory effects on the immune system [84]; and (4) probiotics may influence intestinal homeostasis via the production of their metabolites (e.g., SCFAs) and their interaction with the enteric nervous system [85]. The supplementation of Lactobacillus has been shown to reduce complication rates and shorten hospital stays, indicating less requirement for surgical intervention [86]. In addition, the efficacy of probiotics alone or in combination with two antibiotics was explored in AP mouse models. Probiotics reduced pathogenic bacteria translocation and the combination of probiotics with antibiotics reduced histopathological scores and oxidative parameters, suggesting that probiotics combined with antibiotics have better efficacy than probiotics alone in reducing pancreatic damage [87]. In clinical practice, many issues are associated with probiotics utilization, including the drug resistance of probiotics, hypersensitivity reactions caused by probiotics, probiotic-induced infections, and sepsis. Anti-infective treatment is more difficult when potentially pathogenic bacteria turn to pathogenic bacteria. In terms of experimental design, size, quality control, and study population, the roles of probiotics in AP are still controversial [88]. Further studies are needed to describe the effect of probiotic interventions and understand the mechanisms of probiotics on AP therapies.

### 4.2. Antibiotics

Antibiotics can inhibit the growth of pathogenic organisms in patients with SAP and reduce the complications of secondary infections. However, the overuse of broad-spectrum antibiotics has led to the appearance of multi-resistant strains and increased mortality in AP mice [89]. The combination of vancomycin, neomycin, and polymyxin B was able to prevent the progression of experimental AP to severe disease in mouse models, suggesting that targeting specific bacteria could be beneficial for AP treatment [51]. In addition, selective digestive decontaminants (SDD) could decrease the incidence and mortality of infection-related complications in animal models and SAP patients [12,90]. Currently, broad-spectrum antibiotics are not recommended for AP treatment while targeting specific pancreatic flora or gut pathogenic bacteria with narrow-spectrum antibiotics may improve the outcome.

### 4.3. FMT

Fecal microbial transplantation (FMT) is a therapeutic strategy in which fecal material from healthy donors is implanted into the patient’s gut to restore gut dysbiosis, which is recommended as a therapeutic strategy for some diseases [91,92,93]. FMT is an effective treatment for several diseases, including UC [94], IBS [95], and hepatic encephalopathy [96]. Nevertheless, the role of FMT in AP treatment has not been clarified. Berg et al. [12] found that FMT resulted in a significant increase in pathogenic bacterial translocation and mortality in AP mice, while Yu et al. [17] found that intestinal mucosal damage in AP mice was significantly relieved by FMT, with the reduced infiltration of the inflammatory cells and increased secretory IgA concentrations. We found that FMT increases plasma nicotinamide mononucleotide (NMN) levels and mediated SIRT3 to improve mitochondrial function to modulate ROS levels and alleviate AP progression in mouse models [97]. The main differences may be caused by a different FMT protocol, namely “off-target transplants”. Thus, studying the alteration of gut microbiota in AP patients and screening for beneficial strains may improve the effect of FMT. In addition, the identification and transplantation of genetically engineered bacteria are expected to improve the effect of bacteria therapy.

### 4.4. Enteral Nutrition

Enteral nutrition (EN) is an efficient way to relieve AP. This protective effect can be traced to specific substances, such as glutamine, arginine, and n-3 fatty acids, which regulate gut microbiota and thus stabilize intestinal function and mucosal barrier [98]. The early application of EN prevents intestinal barrier damage and reduces the rate of bacterial translocation, which reduces the incidence of infectious complications and shortens the length of the hospital stay [99,100]. Jin et al. [101] found that the early supplementation of bifidobacterial-containing EN reduced bacterial translocation and alleviated inflammation, thereby shortening the length of hospital stay for AP patients. Prebiotics are non-digestible components that protect the intestinal barrier, modulate the intestinal microbiota, and alleviate bacterial translocation, which are considered effective treatments for AP. Supplementation with specific dietary fiber components is considered a potentially viable treatment strategy for SAP [53,102]. Studies have shown that supplementing a fiber-rich diet could inhibit the systemic inflammatory responses and alleviate SAP by affecting the diversity of the gut microbiota and increasing the production of SCFAs [103]. In addition, supplementation with pectin-rich EN affects intestinal barrier function [104,105], alters the ratio of *Firmicutes* to *Bacteroidetes* in the gut and reduces inflammatory storm and the disease severity in animal experiments [106,107]. EN, particularly in combination with prebiotics, is able to modulate gut microbiota, protect the intestinal barrier, and inhibit bacterial migration to improve the prognosis of AP patients [108].

### 4.5. Supplementation of Metabolites

Metabolite supplementation is a potential strategy for treating SAP. Butyrate supplementation reduces mortality and alleviates pancreatic injury by strengthening the gut barrier and reducing bacterial translocation in SAP mice [12]. Butyric acid reduces systemic inflammation by inhibiting the STAT1/AP1 signaling pathways in peritoneal macrophages, as well as the secretion of NLRP3 inflammasomes and pro-inflammatory factors [54]. Similarly, acetate alleviates the inflammatory response of the organism by exerting its antibacterial, anti-inflammatory, and antioxidant properties [109,110]. In addition, valproic acid attenuates AP by reducing myeloperoxidase activity and the local tissue destruction of other organs [111]. Nevertheless, the treatment of AP by direct supplementation of metabolites is currently limited to animal studies and still lacks sufficient clinical evidence.

### 4.6. Difficulties and Controversies in Microbiota-Based Treatment

Despite the promising future of microbiota-based therapy strategies, treatment regimen development has been slow and elusive, and clinical trials have not achieved satisfactory results like those obtained from animal studies. Some intervention strategies may even have a negative impact on the treatment of AP. The reasons for this controversy are multifactorial. Kotzampassi et al. [112] believe that the theoretical risks of using probiotics include toxicity, metabolic effects, immune effects on drug resistance gene transduction, and translocation from the gut. Explaining the opposite therapeutic effect of probiotics on AP requires the consideration of several factors, including the timing of initiation of treatment, the course of treatment, the type and dose of probiotics, and individual differences [77]. The use of antibiotics is also currently controversial, with most studies not supporting the prophylactic use of antibiotics in SAP patients. Soares et al. [89] found that multidrug-resistant (MDR) strain translocation caused by the abuse of broad-spectrum antibiotics increased mortality in AP mice. However, with the development of microbiota research, certain selective antibiotics can reduce the incidence of infection complications and mortality in animal models and SAP patients [12,90]. Other therapeutic strategies that directly or indirectly alter the gut microbiota, such as FMT and enteral nutrition, have not shown significant positive effects in clinical studies of AP. Therefore, gut microbial-based treatment strategies for AP still require further research, and future research may focus on finding specific strains that are critical to AP or other metabolites associated with AP. Targeting the gut microbiota may be a potential therapeutic option for AP, but large-scale, multi-center trials are required to further explore the optimal regimen of probiotics, prebiotics, FMT, or enteral nutrition for AP.

## 5. Conclusions and Perspectives

Current evidence improves the understanding of interactions between gut microbiota and the pancreas, and gut microbiota and pancreatic tissue-resident microbes differ in AP patients with different etiology, severity, and complications. Further studies are moving beyond descriptions into mechanistic approaches which tighten the relationships between AP progression and gut microbiota dysbiosis. These cover intestinal barrier disruption, local and systemic immune activation, bacterial migration, and retention, as well as bacteria-derived metabolites. Recent studies have provided promising messages for considering probiotics, antibiotics, FMT, bacterial metabolites, and early enteral nutrition in clinical interventions. Further steps are expected to understand the migration and colonization of bacteria within the pancreas, the interaction of the microbes with the local environment of the pancreas, and intestine and the effect of bacteria on different cell types (e.g., intestinal epithelial cells, immune cells, acinar cells, etc.). The developed techniques such as metagenomics, metabolomics, metatranscriptomics, and culturomics will result in breakthroughs for microbial research and microbial-based therapies.

## Figures and Tables

**Table 1:** Summary of gut microbiota alterations in AP patients.

Study	Reference	Type of Sample	Subject	Microbial Evaluation	Phylum Level	Genus or Species Level
Tan et al. (2015)	[10]	Feces	AP patients vs. Healthy volunteers	PCR-DGGE	NA	*Bifidobacteria* ↓ *Enterobacteriaceae* ↑ *Enterococcus* ↑
Zhang et al. (2018)	[11]	Feces	AP patients vs. Healthy volunteers	16s	*Firmicutes* ↓ *Actinobacteria* ↓ *Bacterodetes* ↑ *Proteobacteria* ↑	NA
Van den Berg et al. (2021)	[12]	Pancreas and intestinal tissue	AP patients vs. Healthy volunteers	16s	*Verrucomicrobia* ↑ *Firmicutes* ↑	*Akkermansia muciniphila* ↑ *Escherichia-Shigella* ↑ *Erysipelotrichaeceae* ↑ *Lachnospiraceae* ↓ *Rumminococcaceae* ↓
Chen et al. (2017)	[15]	Feces	AP patients vs. Healthy volunteers	16s	*Saccharibacteria* ↓ *Tenericates* ↓	*Escherichia-Shigella* ↑ *Phascolarctobacterium* ↑ *Candidatus_Saccharimonas* ↓ *Lachnospiraceae* ↓ *Ruminiclostridium* ↓ *Prevotellaceae* ↓
Li et al. (2013)	[16]	Peripheral blood	AP vs. SAP	PCR-DGGE	NA	*Escherichia coli* ↑ *Enterococcus faecium* ↑ *Bacillus coagulans* ↑
Yu et al. (2020)	[17]	Rectal swab	AP vs. SAP	16s	NA	*Enterococcus* ↑ *Escherichia-Shigella* ↑ *Eubacterium hallii* ↓
Ciocan et al. (2018)	[18]	Feces	alcoholic AP vs. Alcoholic	16s	*Proteobacteria* ↑ *Bacteroidetes* ↓	*Klessiella pneumoniae* ↑ *Lactobacillus* ↑ *Enteraoccus* ↑ *Sphingomonas* ↑
Philips et al. (2019)	[19]	Feces	alcoholic AP vs. Healthy volunteers	16s	*Actinobacteria* ↑ *Bacteroidetes* ↓	*Bacteroides inmobilize* ↑ *Moraxella* ↑
Hu et al. (2021)	[20]	Feces	HTGP vs. AP	16s	*Firmicutes* ↑ *Lachnospiraceae* ↓ *Bacteroidaceae* ↓	*Finegoldia* ↑ *Enterococcus* ↑ *Escherichia-Shigella* ↑ *Bacteroides ovatus* ↓ *Blautia wexlerae* ↓ *Dorea longicatena* ↓
Li et al. (2023)	[21]	Feces	HTGP vs. Healthy volunteers	16s	*Firmicutes* ↑ *Proteobacteria* ↓	*Enterococcaceae* ↑ *Escherichia Shigella* ↑ *Bacteroides* ↓ *Faecalibacterium* ↓
Zhu et al. (2019)	[22]	Feces	SAP vs. AP	16s	*Proteobacteria* ↑ *Bacteroidetes* ↓	*Escherichia–Shigella* ↑ *Enterococcus* ↑ *Enterobacteriaceae* ↑ *Prevotella_9* ↓ *Faecalibacterium* ↓
Zou et al. (2022)	[23]	Feces	NP vs. Non-NP	16s	*Enterobacteriaceae* ↑ *Bacteroidetes* ↓	*Enterococcus facecalis* ↑ *Finegoldia magnas* ↑

↑: increased levels; ↓: reduced levels; AP: acute pancreatitis; NP: necrotic pancreatitis; SAP: severe acute pancreatitis; HTGP: hypertriglyceridemic pancreatitis; PCR-DGGE: polymerase chain reaction denaturing gradient gel electrophoresis; 16s: 16s rRNA gene amplicon sequencing; NA: not available.
